# An Improvement Strategy for Indoor Air Quality Monitoring Systems †

**DOI:** 10.3390/s23083999

**Published:** 2023-04-14

**Authors:** Claudio De Capua, Gaetano Fulco, Mariacarla Lugarà, Filippo Ruffa

**Affiliations:** DIIES—Department of Information Engineering, Infrastructure and Sustainable Energy, Mediterranea University of Reggio Calabria, 89122 Reggio Calabria, Italy; decapua@unirc.it (C.D.C.); gaetano.fulco@unirc.it (G.F.); mariacarla.lugara@unirc.it (M.L.)

**Keywords:** IoT, sensing, monitoring systems, IAQ, air quality, decision making

## Abstract

Air quality has a huge impact on the comfort and healthiness of various environments. According to the World Health Organization, people who are exposed to chemical, biological and/or physical agents in buildings with low air quality and poor ventilation are more prone to be affected by psycho-physical discomfort, respiratory tract and central nervous system diseases. Moreover, in recent years, the time spent indoors has increased by around 90%. If we consider that respiratory diseases are mainly transmitted from human to human through close contact, airborne respiratory droplets and contaminated surfaces, and that there is a strict relationship between air pollution and the spread of the diseases, it becomes even more necessary to monitor and control these environmental conditions. This situation has inevitably led us to consider renovating buildings with the aim of improving both the well-being of the occupants (safety, ventilation, heating) and the energy efficiency, including monitoring the internal comfort using sensors and the IoT. These two objectives often require opposite approaches and strategies. This paper aims to investigate indoor monitoring systems to increase the quality of life of occupants, proposing an innovative approach consisting of the definition of new indices that consider both the concentration of the pollutants and the exposure time. Furthermore, the reliability of the proposed method was enforced using proper decision-making algorithms, which allows one to consider measurement uncertainty during decisions. Such an approach allows for greater control over the potentially harmful conditions and to find a good trade-off between well-being and the energy efficiency objectives.

## 1. Introduction

Nowadays, the culture of design is changing, there is a growing need for a renewed relationship between technology and the environment; therefore, the “digital revolution” is generating major changes in the philosophy of building design. The use of intelligent devices that read and monitor some key parameters to evaluate the performance of buildings is ever more widespread, with the aim of achieving conditions of well-being and safety and also developing strategies that employ the maximum possible energy saving [1,2]. 

It is well known that most of the Italian building stock was built before 1990 when there were no health and hygiene regulations [3], and the climatic context was certainly different from the current one [4]. Moreover, the mediocre performance of building envelopes and the conditions of livability and healthiness inside confined spaces are not sufficient to guarantee elevated levels of quality of life for occupants.

Today, more than ever, the scientific debate is focused on the need to intervene with “internet-addicted” solutions on the built heritage and infrastructure [5] to improve the quality and sustainability of life. 

Focusing on healthiness, recent studies [6,7] have shown that indoor environmental factors (temperature, humidity, indoor ventilation, pollutants, etc.) could have a significant influence on health, especially on the transmissibility of infections. This makes it imperative to renovate buildings, especially considering the estimate of the IEQ (indoor environment quality) with the help of advanced IoT (Internet of Things) systems, which allow the elements of the physical world to be connected online to their digital twins, which are continuously updated with data read in real time by sensors [8,9,10]. To improve the IEQ level, it is important to analyze the concentration of pollutants in the air, and to evaluate the thermal, acoustic and visual comfort in the internal environment [11,12]. Several works have shown that elevated temperatures are associated with a higher prevalence of sick building syndrome (SBS) symptoms among building’s occupants. Keeping the temperature within certain limits positively affects the productivity of the building occupants, as only when people feel thermally comfortable can they produce in full capacity. Thermal comfort plays a key role in productivity, and the best conditions of working and living indoors are created with stable temperatures between 21 and 25 °C [13]. Another parameter to take into account is the relative humidity (RH), which refers to the moisture content in the air. This crucial factor could affect the thermal comfort in a building. There is a significant contribution of dry indoor air in both disease transmission and poor building occupant health. During cold winters, outdoor air is drawn indoors and then heated to a comfortable temperature. This process will significantly lower indoor RH, which creates an extremely dangerous situation for indoor occupants. When indoor RH is less than 40%, humans become more vulnerable to viral respiratory infections [14,15]. Earlier studies have shown that for human health, a relative humidity between 40 and 60% is optimum [16].

Multiple scientific research publications acknowledge that respiratory diseases mainly transmit from human to human through close contact, airborne respiratory droplets and contaminated surfaces. Several studies, moreover, state that there is a relationship between air pollution and spread in terms of the transmissibility of diseases; therefore, accurate monitoring and control of these environmental conditions can be useful in preventing the spread of diseases [17]. 

There are many health problems linked with the IAQ (indoor air quality) level; according to the World Health Organization (WHO), SBS affects people who are subjected to prolonged exposure to chemical, biological and/or physical agents in buildings with low IAQ and poor ventilation [18]. It is known, in fact, that air quality levels are generally two- to five-times worse indoors than outside [19,20,21]. As a result, occupants in confined spaces who do not enjoy good natural (and/or mechanical) ventilation are at an increased risk for psycho-physical discomfort, which, over time, often leads to devastating effects, such as the onset of respiratory tract infections, central nervous system diseases and even cancer. Since lifestyles have changed considerably in recent years, increasing the amount of time spent indoors (by around 90%), monitoring IAQ has become essential, mainly in environments where several people are present at the same time [7,22]. In this scenario, several researchers and ICT (Information and Communication Technology) technicians are investigating how multi-sensor IoT platforms can be used to optimize IAQ levels in buildings through ‘smart ventilation’ systems [23,24]. On the other hand, there is a growing interest in improving energy-saving strategies, to save money and for environmental reasons.

To find a trade-off between these two objectives that can require, in many situations, opposite strategies, the authors propose an innovative monitoring and control algorithm, which considers different user needs, pollutant safety thresholds set by current regulations and, only when necessary, activates ventilation systems to restore air quality levels. The system consists of a hardware platform, connected to the cloud, which acquires and processes sensors data to obtain proper indices, which carry information on both the IAQ level and on the outdoor air quality. Through a ventilation control algorithm, it is possible for the system to operate both autonomously and remotely controlled [25], to improve indoor comfort levels and energy saving. Ventilation, that is, the process of providing outdoor air in a space or building through natural or mechanical means, plays a critical role in removing exhaled virus-laden air, thus lowering the overall concentration and, therefore, any subsequent dose inhaled by the occupants [26]. By adjusting the ventilation of the indoor environment, it is possible to reduce the CO_2_ level [27,28], reduce the concentration of PM (particulate matter) and maintain a constant humidity level [29], guaranteeing a healthier environment with less possibility of disease transmission [30]. Furthermore, to improve efficiency and reliability, the algorithm uses decision-making algorithms to consider the uncertainty related to measurement data, a key factor that cannot be neglected in situations in which a comparison between measured values and a set threshold is required. Using the decision-making algorithms, it is possible to know a priori the probability that the threshold is really crossed and, on this basis, to make a decision on the actions to be taken.

The main contributions of the proposed monitoring algorithm to the state of the art in IAQ monitoring systems consist of the integration of new indices able to take into account the duration of exposure to pollutants and the use of decision-making algorithms, allowing one to properly consider measurement uncertainty during the decision-making process.

The document is composed as follows: Section 2 describes the designed monitoring and control station, critically analyzing the choice of monitored parameters through a synthesis of the state of the art in Section 2.1; Section 2.2 reports, in detail, the methodology proposed for the computation of the IAQ index; Section 3 describes two decision-making algorithms, each one explained in detail in Section 3.2.1 and Section 3.2.2, while in Section 3.3, the validation results of the proposed algorithms are reported using some sets of simulated data; Section 4 describes the results obtained with the proposed approach and reports a comparative analysis with the state of the art; Section 5 summarizes the contents and concludes the document.

## 2. Methods and Applications

The proposed design relies on a sensor network, which can be based on either wired sensors or wireless sensors, using the latest communication technologies [31]. 

The transducer section is composed of CO, VOC, CO_2_ and PM2.5 sensors and consists of two parts, dedicated to the detection of internal and external environmental conditions. The technical characteristics of the sensors used in the proposed monitoring and control station are shown in Table 1. These characteristics are used later in the calibration of the decision-making algorithms described in Section 3.

**Figure 1:**
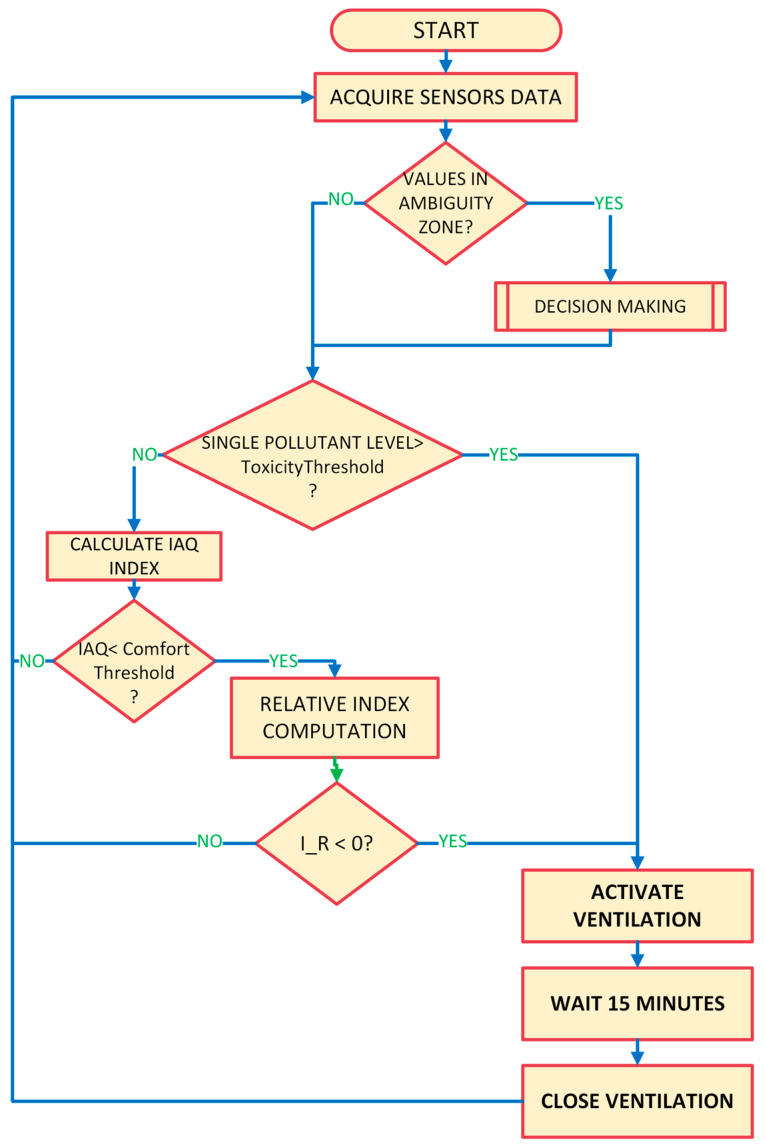
Proposed algorithm flowchart.

The measurement system constantly measures the pollutant level inside and outside the monitored environment. Using the measurement data, the control station tries to maintain a satisfactory level of IAQ, through a forced ventilation system, also considering the quality of the outside air, because it could be lower [32,33] and, in this last case, the opportunity of activating ventilation must be evaluated. When a risky situation is detected from the data analysis and sensor data fusion process, it is necessary to consider all the parameters measured with the right weight on the overall IAQ. For this reason, in this paper, the authors propose the use of new indices, also considering the exposure time. The uncertainty associated with measurements is an important factor in determining if the pollutant level is within the limits; in fact, a set threshold could be crossed because of the uncertainty contribution, or worse, the pollutant level detected could be within the limit even though it exceeded the set threshold. For this reason, decision-making algorithms were integrated into the platform to improve the analysis and management of environmental measurement data. The overall functioning of the algorithm proposed for the automatic monitoring and control station is shown in a flowchart in Figure 1 and, with a higher level of detail in the pseudo-code reported in Listing A1.

### 2.1. Monitored Parameter

As stated in [34], indoor pollutant sources, indoor activities, ventilation rates, as well as outdoor air can affect indoor air quality and so the healthiness and comfort of environments.

The parameters for the evaluation of IAQ levels were selected from a synthesis of the state of the art, considering the current regulations in many states [35]. In many recent works [36,37,38,39,40,41,42], according to green building schemes and some standards summarized in Table 2, the focus was on three commonly studied air pollutants: carbon dioxide (CO_2_), total volatile organic compounds (TVOCs) and particulate matter smaller than 2.5 μm (PM2.5).

CO_2_, in closed environments with limited ventilation, can reach values around 5000 ppm and 6000 ppm, but the limit of 1000 ppm of CO_2_ must not be exceeded to have good air quality [43]. CO_2_ is often an indicator for the effectiveness of ventilation and excessive population density in a structure. Examining levels of CO_2_ in indoor air can provide information regarding occupant densities and outdoor air ventilation rates [21]. Another pollutant parameter analyzed is particulate matter (PM2.5 and PM10), considering the relationship between the concentration of these atmospheric pollutants and the transmission of a respiratory virus [44]. The PM concentration in the air is directly associated with respiratory diseases and infections such as asthma, as well as cardiovascular and respiratory diseases, including lung cancer [45]. To evaluate the concentration of PM2.5 and PM10, the reader can refer to the table provided by the Victoria EPA Institute (Australia) [46], which shows the categories obtained based on the hourly and 24 h moving average concentration of PMs. Volatile organic compounds (VOCs) are commonly found in homes. They are generated by household products in residences, followed by combustion processes and environmental tobacco smoke, deodorizers and off-gassing of building materials [47]. These chemicals can cause irritation to the eyes or nose, dizziness, nausea, headaches and allergic reactions, and some of them are carcinogenic [48]. The VOC concentration can be considered an important parameter for the evaluation of IAQ [41].

Furthermore, the inclusion of the CO level in the measurement of IAQ is proposed, since it is an odorless, colorless and very toxic gas, which can be generated indoors by, for example, unvented kerosene and gas space heaters, leaking chimneys and furnaces, back-drafting from furnaces, gas water heaters and other sources.

### 2.2. IAQ Index Calculation

In [34], the IAQ level is defined as the lowest level of satisfaction with one of these parameters, as shown in Equation (1).

(1) $$I_{IAQ}=min(I_{CO2},I_{TVOC},I_{PM2.5})$$

where I_CO2_, I_TVOC_ and I_PM2.5_ are the quality indices of CO_2_, TVOC and PM2.5, calculated as shown:

(2) $$I_{X}=100-\alpha \log\frac{C_{X}}{C_{X}{|}_{0}}$$

where $C_{X}$ is the measured quantity of a generic pollutant, $C_{X}{|}_{0}$ is the concentration of the same pollutant in ideal conditions and α is a constant that considers the toxic levels of the pollutant defined by the current regulations, so that if C_X_ reaches the set threshold, $I_{X}=0$. This method allows one to obtain global and rapid information on the air quality level in indoor environments and can be easily implemented without much computational effort. Further, for a generic pollutant, the quality index, calculated as shown in Equation (2), does not consider the exposure time and its effects on health and comfort. It is well known in the literature that the impact of a toxic substance on human health depends both on its concentration and on the exposure time, so that a concentration that is within the normal range, considering an exposure time of 15 min, would be toxic if the exposure time rises to 1 h or more. As an example, calculating the quality index for CO_2_ with Equation (2), considering a toxic level of 15,000 ppm (EU regulations, toxic level considering an exposure time of 15 min) and a baseline level of 415 ppm, we have α=64.8. If the measured CO_2_ in the air is 1000 ppm, from Equation (2), we obtain $I_{CO2}=75.25$, which is true if the exposure time is 15 min but not if the exposure time is 24 h. In fact, in this case, the index should be zero. 

In this paper, the authors propose a new method for measuring the quality indices of single pollutants, considering both their concentration and the exposure time, as shown in Equation (3):

(3) $$I_{X}=100-\alpha (t)\log\frac{\int_{t0}^{t1}C_{X}dt}{\int_{t0}^{t1}{C_{X}|}_{0}dt}$$

where α(t) adjusts the index value, considering the various thresholds set for different exposure times. For regulations from different states that define different thresholds for the same exposure time, to guarantee maximum safety, the lowest value was considered. The value of α(t) was obtained supposing that $I_{X}=0$ in the risk conditions [34] and performing a quadratic fit between the known points, so that:

(4) $$\alpha \left(t\right)=\alpha_{0}+\alpha_{1}t+\alpha_{2}t^{2}$$

The $\alpha_{0},\alpha_{1},\alpha_{2}$ values obtained for the different pollutants considered are summarized in Table 3, and in Table 4, the baseline concentrations ${C_{X}|}_{0}$ considered for each pollutant are reported.

Therefore, in the system proposed, the IAQ level is measured as:

(5) $$I_{IAQ}=\min\left({I_{CO},I}_{CO2},I_{TVOC},I_{PM2.5}\right)$$

However, in some cases, it is not possible to know a priori if it is a good idea to open the windows and let the outside air enter, even though the IAQ is below the set threshold. As an example, if there is a fire in the area, or a leak of pollutants from a factory or high concentrations of smog in the city center, the outside air quality could be even lower than the indoor one. If this is the case, opening doors and windows could only worsen the situation. For this reason, in this paper, we propose an evaluation of relative indices, which rely on the measurement of pollutants both indoors and outdoors, as shown in Equation (6), to determine if the outdoor air quality is better than that measured indoor quality. Since, in this case, we need to consider the situation in a particular moment and not over a long time span, the integral measurement can be limited to the last 15 min.

(6) $$I_{R\_X}=\log\frac{\int_{t0}^{t1}{C_{X}|}_{indoor}dt}{\int_{t0}^{t1}{C_{X}|}_{outdoor}dt}$$

(7) $$I_{R\_IAQ}=min({I_{R\_CO},I}_{R\_CO2},I_{R\_TVOC},I_{R\_PM2.5})$$

From Equations (6) and (7), it can be seen that if the relative air quality index is greater than zero, it is always a good idea to change the air.

## 3. Data Uncertainty and Decision Making

### 3.1. Data Uncertainty and Decision-Making Problems

**Figure 2:**
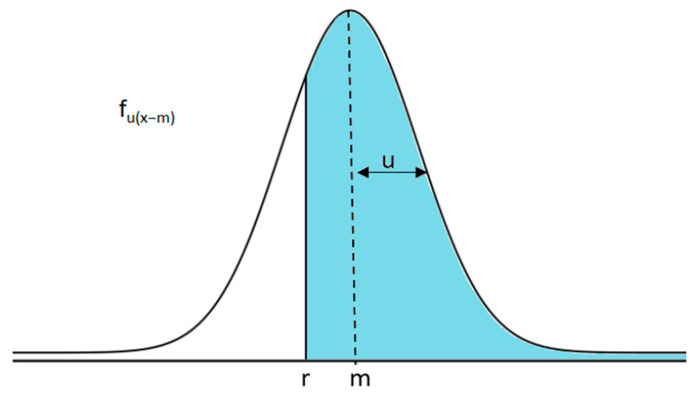
Distribution of the measurement result around the nominal value m.

In comparing the measured environmental parameters with the threshold values established by the relevant regulations, it is particularly important to take into account the measurement uncertainty in the various sensors distributed in the environment. In fact, measurement uncertainty plays a primary role; the result of a measurement provides an interval, centred in the measured value, in which it is possible to find the value of the measurand with a given level of confidence [49,50]. The comparison operation, therefore, must be performed between a single value, the threshold and a range of values, the measurement result. If u(x) is the measurement uncertainty obtained and assuming that it has a normal distribution, it is possible to calculate the associated probability density function (pdf) f_u(x)_. Thus, f_u(x − m)_ represents the pdf of the measurement result around the measured nominal value m, as shown in Figure 2. At this point, the probability P_R_ that the measured value is higher than the threshold r can be expressed as in Equation (8):

(8) $$P_{R}=P[x>r]=\int_{r}^{+\infty }f_{u\left(x-m\right)}dx$$

The risk that the measurand exceeds the defined threshold is graphically expressed as the area subtended by the pdf between the limits of the integral. Once the threshold X_TH_ has been fixed, considering the expanded uncertainty U(x) associated with the measured parameter x, it is possible to define a confidence interval X_TH_ ± U(x), which allows one to determine if the measured value is above or below the threshold with a given level of confidence P, if the probability density function *p(x)* is known. If the measurement result is lower than X_TH_ − U(x), the parameter under control can be considered within the specifications with a certain probability P; if it is greater than X_TH_ + U(x), it will always be non-compliant with probability P [51,52]. 

**Figure 3:**
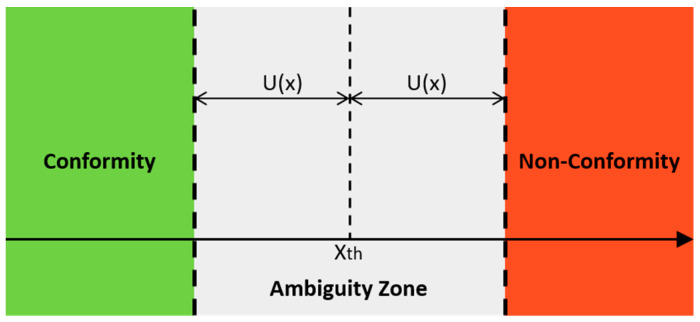
Conformity zone and non-conformity zone.

Further considerations should be made if the measure falls into the so-called ambiguity zone, graphically defined in Figure 3:

X_TH_ − U(x) ≤ x ≤ X_TH_ + U(x) (9)

It is possible to reduce the width of this band of ambiguity in order to decrease the probability of falling into it but, once the measurement system has been characterized, this means choosing a smaller coverage factor, which, therefore, decreases the correctness of the decision. When our measurement falls within the region of ambiguity, there are two decision alternatives, I0 if the measurement result belongs to the compliance zone A or I1 if it belongs to the non-conformity zone R. This is a particular class of problems, called decision-making problems, in which the final goal is the weighted and justified assumption of a decision among several options [53,54,55]. In the specific case of this application, where the result of the measurements is used to deal with the problem of verifying the compliance of the parameters under analysis with a reference (given by regulations or technical specifications), the decision-making algorithms mentioned above are particularly useful. Considering that exceeding the set thresholds causes an activation of the actuators, aimed to restore the optimal environmental parameters, the implementation of such algorithms not only increases the reliability of the measurement but also improves the energy-saving effort. To analyse the acquired data and verify the correctness of the value of the single sensor, considering the measurement uncertainty, two decision-making methods have been developed: the Utility Cost Test [56,57,58] and the Fixed Risk [59,60].

### 3.2. Decision-Making Algorithms

#### 3.2.1. Utility Cost Test

According to the Utility Cost Test [61,62,63], the approach followed to make a decision in conditions of uncertainty considers the consequences that this decision could entail. These consequences are referred to as utilities. Therefore, the utilities of all possible situations must be defined in order to decide whether the value exceeds the threshold or not, taking into account measurement uncertainty, defining:

- u_++_ when the value is below the threshold and is correctly classified as such;
- u_+−_ when the value is below the threshold and is incorrectly classified above

the threshold;

- u_−+_ when the value is above the threshold and is incorrectly classified below

the threshold;

- u_−−_ when the value is above the threshold and is correctly classified as such,

with u_++_ > u_+−_ e u_−−_ > u_−+_.

The decision mechanism can be interpreted as a binary problem. We divide the space of the solutions S into two parts: A (acceptance, sub-threshold) and R (rejection, over the threshold). If the result of the measurements relating to the test falls into A, the data are considered compliant with the requirements.

The utility u_A_ corresponding to the decision of accepting the sub-threshold value hypothesis is:

(10) $${u_{A}=p}_{A}\cdot u_{--}+p_{R}\cdot u_{-+}$$

The utility u_R_ corresponding to the decision of not accepting the sub-threshold value hypothesis (over the threshold value) is:

(11) $${u_{R}=p}_{A}\cdot u_{+-}+p_{R}\cdot u_{++}$$

Based on this criterion, the hypothesis of sub-threshold value is accepted if u_A_ ≥ u_R_, that is, when:

(12) $$\begin{array}{c} p_{A}\cdot u_{--}+p_{R}\cdot u_{-+}\geq p_{A}\cdot u_{+-}+p_{R}\cdot u_{++}\\ p_{A}\left(u_{--}-u_{-+}-u_{+-}{+u}_{++}\right)\geq u_{++}-u_{-+} \end{array}$$

that means, for the assumptions made:

(13) $${(u}_{++}-u_{+-})+\left(u_{--}-u_{-+}\right)>0$$

In conclusion, the measured value will be considered sub-threshold if: $p_{A}\geq p^{\left(0\right)}$ with:

(14) $$p^{\left(0\right)}=\frac{u_{--}-u_{-+}}{u_{++}-u_{-+}-u_{+-}{+u}_{--}}$$

Knowing the measurement uncertainty in the sensor, if its measures are characterized by a normal distribution, it is possible to set the appropriate utilities. The algorithm will then be able to take a decision on exceeding the threshold within the area of ambiguity and to give an answer to the decision-making problem, together with a quantification of the risk associated with the decision taken. A pseudo-code of the Utility Cost Test implementation is reported in Listing A2.

#### 3.2.2. Fixed Risk

**Figure 4:**
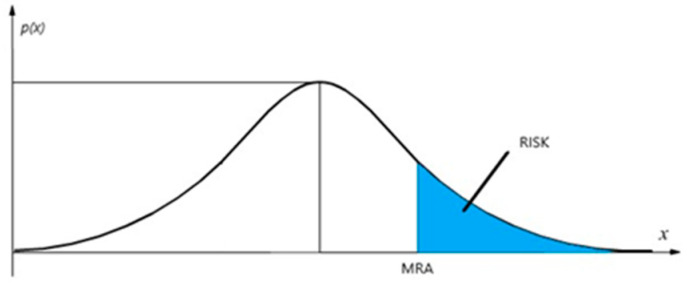
Risk Level.

According to this method, however, a maximum level of admissible risk of making an incorrect decision must be established a priori:

Once an X_TH_ threshold has been set, by comparing the measurement result with it, it is observed that, if the measured value falls within the range of ambiguity, there is a risk, as highlighted in Figure 4 (blue area), of considering the measured value below the threshold when it is actually above. Since the probability density function associated with the measure is known, it is possible to set the maximum risk that is considered acceptable (MRA) in making the decision and, through a change of variable, the threshold is repositioned so that the following relationship is verified:

$$MRA=\int_{Xmra}^{+\infty }f_{u\left(x-m\right)}dx$$

where $f_{u\left(x-m\right)}$ is the probability density function centered in m. A pseudo-code of the Fixed Risk implementation is reported in Listing A3.

### 3.3. Validation of Decision-Making Algorithms

In the field of indoor air quality monitoring, the choice of using decision-making algorithms is dictated by the need to make the system as reliable as possible. The intervention of the forced ventilation system is derived from the evaluation of the values detected by the single sensors used and from the comparison of these values with the predetermined thresholds. We decided to use two reference thresholds. The first is linked to the sector regulations that indicate the toxicity values of a particular pollutant, and the second, less stringent, is linked to the level of comfort that you want to guarantee in the controlled environment. In any case, since sensors and measurements are affected by uncertainty, they always fall within the scope of decision-making problems. Two different decision-making algorithms were implemented to support the decision-making process, and these algorithms were subsequently characterized to respond to two different management strategies:

- Max Air Quality and Healthiness
- Max Energy Saving

**Figure 5:**
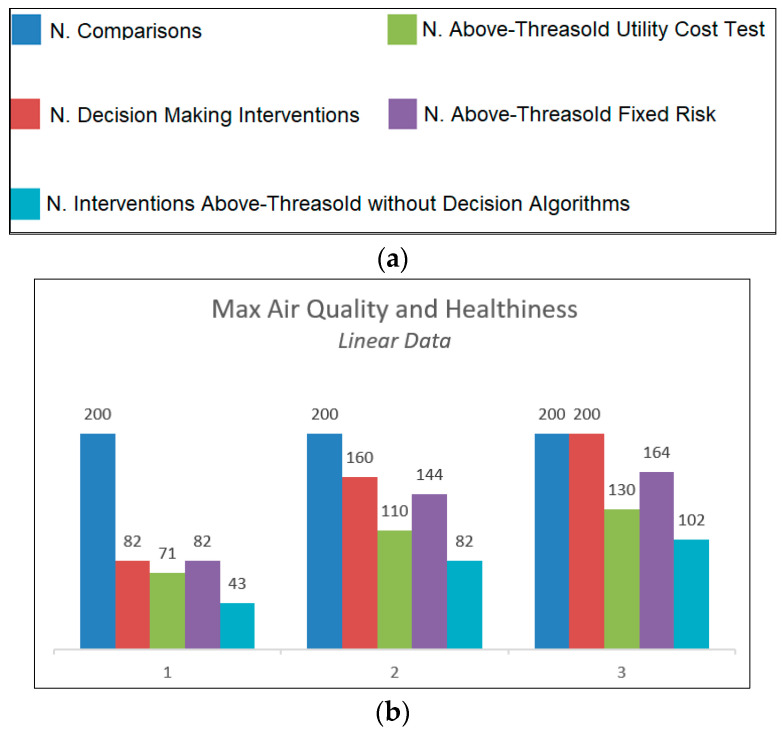

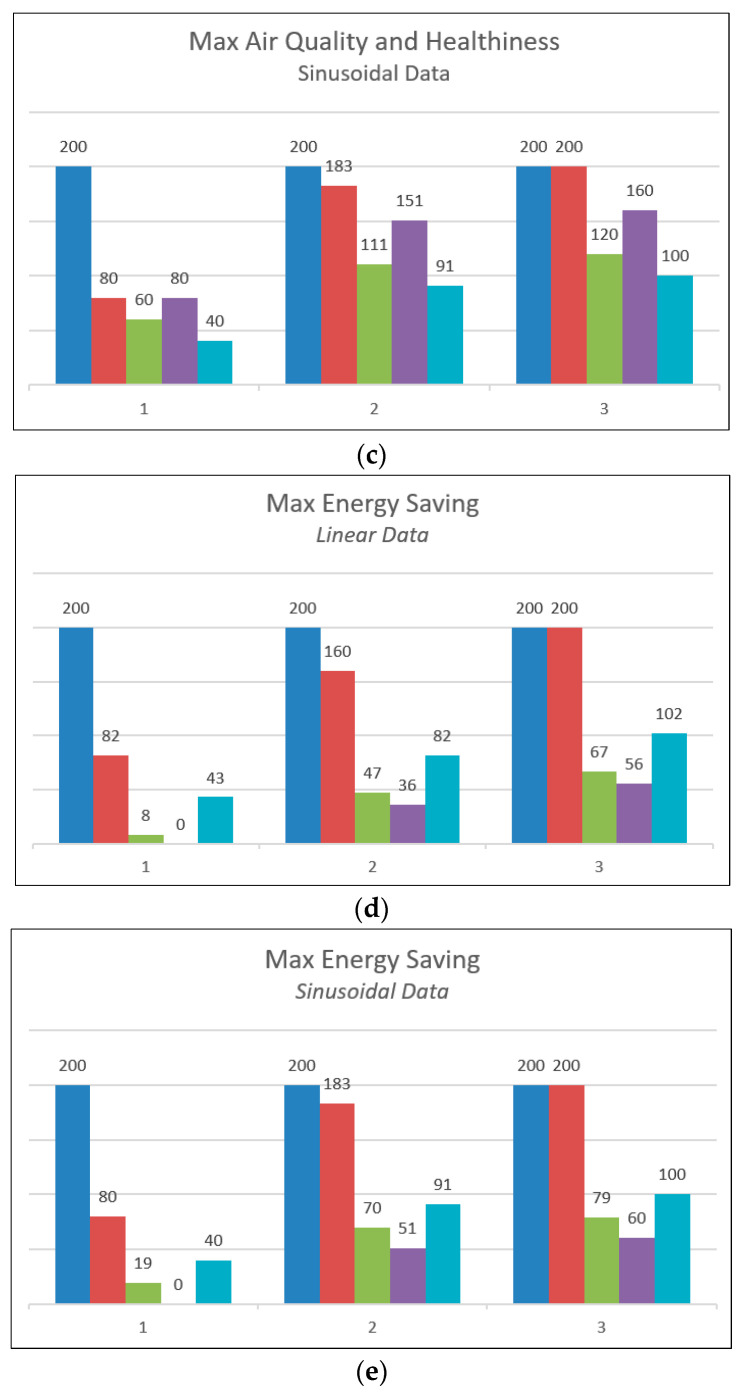
(**a**) Legend. (**b**) Max air quality and healthiness (linear data). (**c**) Max air quality and healthiness (sinusoidal data). (**d**) Max energy saving (linear data). (**e**) Max energy saving (sinusoidal data).

Once the operational strategy is selected in the management system, the system calculates the utilities for the application of the Utility Cost Test and sets the maximum percentage of risk allowed in considering the reference threshold exceeded in Fixed Risk. The two algorithms, calibrated in this way, will take weighted decisions in considering the results of the measurements that fall within the range of ambiguity, above or below the threshold, based on the general strategy chosen. To verify the correct functioning of the algorithms, simulated data sets were used. Considering a generic measured parameter X, which must be compared with a threshold X_TH_, linear variation around the threshold and sinusoidal variation were simulated, maintaining a normal distribution and a predefined standard deviation. The intervention of the algorithms was tested with different coverage factors, and the number of interventions that considered the threshold exceeded was compared with the number of activations that would have occurred without the implementation of the decision algorithms. The results obtained are summarized in the graphs in Figure 5.

From the analysis of the results shown in Figure 5, referring to the two different operational strategies, it can be seen that, if the strategy chosen aims at maximizing air quality and minimizing risks to health, the decision-making algorithms activate ventilation a greater number of times compared to what would have occurred with a direct comparison with the threshold. In the specular case, which instead aims at maximizing energy savings by minimizing the number of activations of the ventilation system, we observe a strong reduction in interventions, about 40–50% less than operating without decision algorithms. Another fundamental aspect is that the response of the algorithms is customizable in environments with different intervention needs by implementing a correct risk assessment in the design phase of the system.

## 4. Results

**Figure 6:**
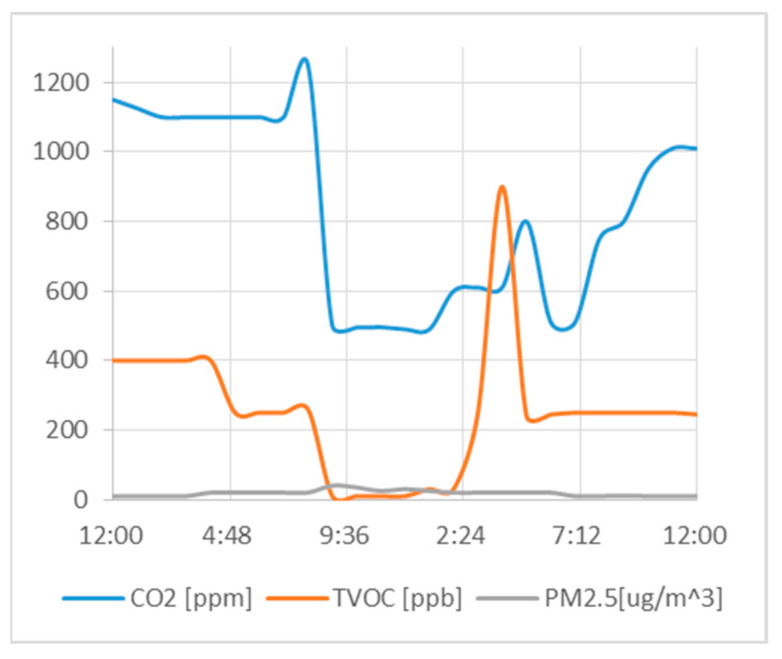
CO_2_, TVOC and PM2.5 in an elderly care facility bedroom over 24 h.

**Figure 7:**
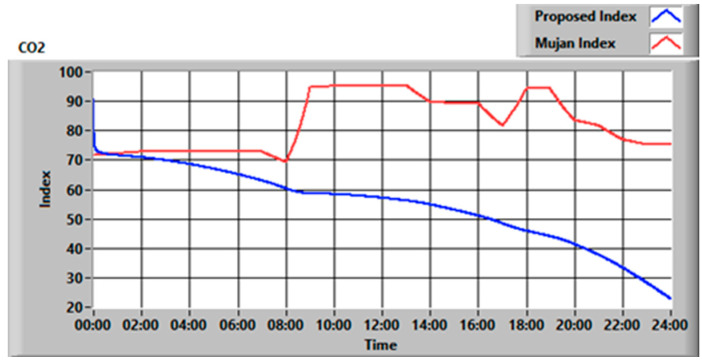
CO_2_ index comparison for the examined case.

To evaluate the impact of the proposed methodology, the same was compared with other recent works. The key innovative points are summarized in Table 5. In particular, the results obtained in this section were compared with the most recent and most complete of the works reported in Table 5, i.e., Mujan et al. [34]. We started testing the algorithm with 24 h real case monitoring data [36], as shown in Figure 6, of an elderly care facility bedroom. Figure 7 shows, as an example, a comparison between the CO_2_ index calculated with the method proposed and the state of the art.

As can be seen in Figure 7, the proposed index drops over time since it considers the exposure time to the pollutant. In this way, after the night, at 8 AM, the proposed index is 10% lower than that calculated with the method proposed by Mujan et al. [34]. Since exposure time plays a key role in the pollutant impact on human health, not considering that aspect could lead to the calculation of falsely high air quality levels. 

**Figure 8:**
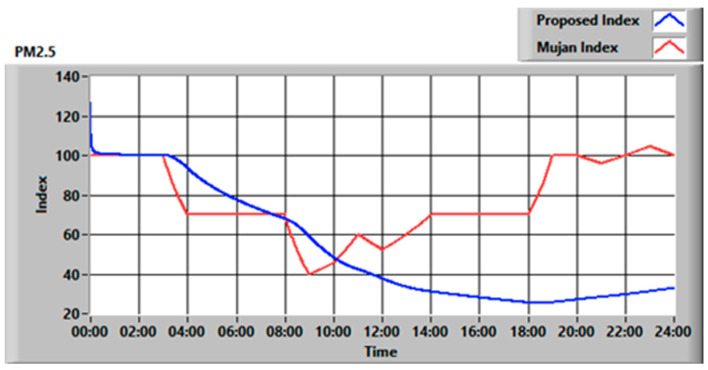
PM2.5 index comparison for the examined case.

On the other hand, as can be seen in Figure 8 for the PM2.5 index, the algorithm proposed is less responsive in the case of fast rises in pollutant concentration. This, in many cases, is not significant, but in rare situations, when the substance rapidly reaches extremely high concentrations, it could be harmful to have a slower detection method. Therefore, in order to guarantee maximum safety, the best solution is to monitor both the cumulative index, which takes into account the exposure time, and the instantaneous index. In an automatic control station, the algorithm should reset the cumulative index every time the air-changing actuator is triggered. The index, in fact, has memory and would result as low, even though the air quality level has been restored. A good trade-off between safety, both over a short and a long time, and computational complexity could be the usage of an average index for each pollutant calculated over a brief period of time, such as 15 min. In this way, the minimum threshold for the average index must be set high enough to guarantee that the cumulative index over 24 h will always be above the safety threshold. Obviously, such an approach has a drawback, since having a higher threshold means more frequent actuator activations and so higher energy consumption.

To validate the proposed technique, considering a certain number of possible trends for the pollutants, the algorithm was tested on a huge set of data generated with the Monte Carlo method. Since the same considerations can be made for all the pollutants, and the objective of this simulation is to validate the algorithms and to compare their behavior with the state of the art, in this paper, only the results obtained for the CO_2_ index are shown.

**Figure 9:**
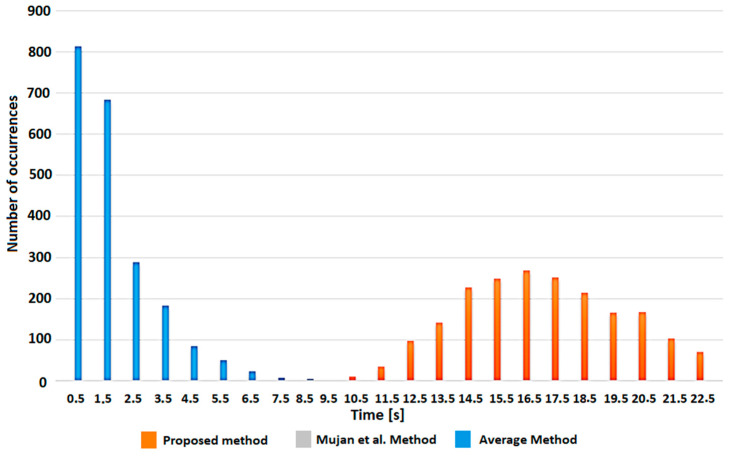
Threshold exceeded with the different algorithms on Monte Carlo simulation data.

The algorithms were tested on 10,000 sets of data obtained from a Monte Carlo simulation, with a starting point of 500 ppm of CO_2_ and a standard deviation of 10 ppm. After discarding the patterns that include impossible values, only 2144 patterns were evaluated. In Figure 9, the results of the test in terms of time needed to trigger the actuators are summarized, considering a minimum threshold for the CO_2_ index of 60%. In the case of the average index method, the threshold was recalculated to guarantee that, in the worst case, the cumulative index over 24 h would be above 60%, so obtaining a new threshold of 90%. As can be seen, with the method used by Mujan et al., the index would almost always be within the limits, and the actuators would rarely be triggered (just four occurrences). On the other hand, with the average index method, the higher threshold causes early, and perhaps more frequent, actuator activation. With the method proposed, it is possible to guarantee a good trade-off between safety requirements and energy consumption.

## 5. Conclusions and Future Perspectives

In this work, the authors proposed an innovative IAQ optimization strategy, which relies on a smart monitoring and control system, which monitors the quality of indoor and outdoor air and makes decisions aimed at optimizing the internal air quality. The control station acquires measurements from a sensor network and uses decision-making algorithms to intervene on the ventilation system to restore the optimal IAQ levels. To improve the state of the art, the authors proposed new indices that consider both the concentration of pollutants and the exposure time to obtain a more realistic assessment of the risks. Furthermore, the use of decision-making algorithms in the control system application increases the reliability of the decisions taken, allowing one to consider the measurement uncertainty when comparing measurement values with thresholds set by regulation standards. This allows one to optimally manage the decision-making process in conditions of uncertainty and personalize the methods of intervention. Two decision-making algorithms have been implemented: the Utility Cost Test and the Fixed Risk. In some critical environments, such as a classroom, it may be preferable to use the Utility Cost Test to quantitatively assess the impact of a possible wrong decision and decide to act more cautiously. In other environments, it can be difficult to identify significant cost functions, and, in these cases, the Fixed Risk test can be used to make decisions with a known level of risk. The user can personalize them for the operative stage, choosing one of the proposed management strategies. Furthermore, to make the system safer and more reliable, if the IAQ is below a set threshold, the algorithm calculates and evaluates a relative index for IAQ, which allows one to make a comparison between the indoor and outdoor air quality to decide if it is the case to change the air.

The algorithm was validated using both real-world data and simulated data with the Monte Carlo method. A comparison between the results obtained using the methodology proposed and the state of the art shows a substantial benefit in terms of safety and energy efficiency. In fact, we observed that with the state of the art, the number of times the system intervenes to restore air quality strongly depends on the set threshold. In this case, depending on the specific data, the system could rarely intervene, or even intervene too early and too frequently, causing a potential harmful situation in one case and unnecessary energy waste in the other. The proposed method, also with the use of decision-making algorithms, showed performances that can guarantee the best trade-off between high safety and energy efficiency requirements.

Future developments will regard the extension of the system for the measurement and evaluation of the IEQ, considering all the aspects related to the thermal, acoustic and visual comfort and the implementation of sensor fault detection strategies to improve reliability and make the system more fault tolerant.

## Figures and Tables

**Table 1:** Technical characteristics of the sensors.

Sensor	Model	Unit	Range	Accuracy
CO	TGS3870	mg/m^3^	50–1000	±20
CO_2_	USEQGCCAC82S00	ppm	0–10,000	±40
VOC	PS1-VOC-1000	ppm	0–1000	±0.1
PM2.5	SPS30	µg/m^3^	0–1000	±10

**Table 2:** Selected parameters and their adoption by standards and green building schemes [34].

Standard	CO_2_	TVOC	PM2.5
BES	Yes	Yes	
BREEAM		Yes	
DGMB		Yes	
EN 16798	Yes		Yes
HQE		Yes	Yes
KLIMA	Yes	Yes	
LEED	Yes	Yes	Yes
NABERS	Yes		Yes
OsmoZ	Yes	Yes	Yes
WELL	Yes		Yes

**Table 3:** α values obtained for the different pollutants.

	α_0	α_1	α_2
**CO_2_**	63.84	0.02	8.04 × 10^−5^
**TVOC**	209.60	0.00	0
**PM2.5**	89.14	0.18	−4.97 × 10^−5^
**CO**	48.58	0.68	−100.36 × 10^−5^

**Table 4:** Ideal concentration of pollutants for excellent IAQ [36].

	${C_{X}|}_{0}$
**CO_2_**	415 ppm
**TVOC**	186 ppb
**PM2.5**	10 µg/m^3^
**CO**	1.7 ppm

**Table 5:** Comparison between the proposed method and others, known at the state of the art, in assessing IAQ level.

	Monitored Parameters	IAQ Level Is Function of Exposure Time	Considers Measurement Uncertainty during Comparison with Reference Threshold
Proposed Methodology	CO_2_, TVOC, PM and CO	YES	YES
Serroni et al. [17]	CO_2_ and PM	NO	NO
Mujan et al. [34]	CO_2_, TVOC and PM	NO	NO
Hapsari et al. [37]	CO_2_ and PM	NO	NO
Pietrogrande et al. [38]	CO_2_, TVOC and PM	NO	NO

## Data Availability

Not applicable.

## Appendix A

The pseudo-code of the algorithm proposed for the automatic monitoring and control station is shown in Listing A1; in Listings A2 and A3, the pseudo-code of the Utility Cost Test and Fixed Risk subroutines is shown.

**Listing A1.** Pseudo-code of the control station algorithm
define COthreshold, CO2threshold,VOCthreshold,PMthreshold; define COuncertainty, CO2uncertainty, VOCuncertainty, PMuncertainty; define alfa0CO, alfa1CO, alfa2CO, alfa0CO2, alfa1CO2, alfa2CO2, alfa0VOC, alfa1VOC, alfa2VOC, alfa0PM, alfa1PM, alfa2PM; define optimalCO, optimalCO2, optimalVOC, optimalPM; define comfortThreshold; define acquisitionRate; setup(){ \* select strategy and set activateVentilation to FALSE (ventilation OFF) strategy=keyboard.in(); decisionMakingType=keyboard.in(); startTime=getTime(); activateVentilation=FALSE; } main(){ **while**(STOP){ previoustime = time; COreading[] = CO.insert(sensorRead(sensor1)); CO2reading[] = CO2.insert(sensorRead(sensor2)); VOCreading[] = VOC.insert(sensorRead(sensor3)); PMreading[] = PM.insert(sensorRead(sensor4)); outdoorCOreading[] = outdoorCOreading.insert(sensorRead(sensor5)); outdoorCO2reading[] = outdoorCO2reading.insert (sensorRead(sensor6)); outdoorVOCreading[] = outdoorVOCreading.insert(sensorRead(sensor7)); outdoorPMreading[] = outdoorPMreading.insert(sensorRead(sensor8)); time = getTime(); maxdimension 900/acquisitionRate; /* number of samples in 15m if(outdoorCOreading.size()>maxdimension){ COreading.delete(0); CO2reading.delete(0); VOCreading.delete(0); PMreading.delete(0); outdoorCOreading.delete(0); outdoorCO2reading.delete(0); outdoorVOCreading.delete(0); outdoorPMreading.delete(0); } \*Calculate cumulative values of pollutants during monitoring interval cumulativeCO=cumulativeCO+TrapezoidalRuleIntegral(COreading.lastElement(), COreading.lastElement()-1, time, previousTime); cumulativeCO2=cumulativeCO2+TrapezoidalRuleIntegral(CO2reading.lastElement(), CO2reading.lastElement()-1, time, previousTime); cumulativeVOC=cumulativeVOC+TrapezoidalRuleIntegral(VOCreading.lastElement(), VOCreading.lastElement()-1, time, previousTime); cumulativePM=cumulativePM+TrapezoidalRuleIntegral(PMreading.lastElement(), PMreading.lastElement()-1, time, previousTime); \*Calculate the optimal cumulative value of pollutants (ideal case) cumulativeOptimalCO=cumulativeOptimalCO+TrapezoidalRuleIntegral(optimalCO, optimalCO, time, previousTime); cumulativeOptimalCO2=cumulativeOptimalCO2+TrapezoidalRuleIntegral(optimalCO2, optimalCO2, time, previousTime); cumulativeOptimalVOC=cumulativeOptimalVOC+TrapezoidalRuleIntegral(optimalVOC, optimalVOC, time, previousTime); cumulativeOptimalPM=cumulativeOptimalPM+TrapezoidalRuleIntegral(optimalPM, optimalPM, time, previousTime); \* if values in ambiguity zone call DecisionMaking subroutine to assign a value to activateVentilation if(COreading.lastElement()>=COthreshold-COuncertainty && COreading.lastElement()<=COthreshold+COuncertainty){ activateVentilation=DecisionMaking(strategy, decisionMakingType, COreading.lastElement(), COuncertainty, COthreshold);} else if(CO2reading.lastElement()>=CO2threshold-CO2uncertainty && CO2reading.lastElement()<=CO2threshold+CO2uncertainty){ activateVentilation=DecisionMaking(strategy, decisionMakingType, CO2reading.lastElement(), CO2uncertainty, CO2threshold);} else if(VOCreading.lastElement()>=VOCthreshold-VOCuncertainty && VOCreading.lastElement()<=VOCthreshold+VOCuncertainty){ activateVentilation=DecisionMaking(strategy, decisionMakingType, VOCreading.lastElement(), VOCuncertainty, VOCthreshold);} else if(PMreading.lastElement()>=PMthreshold-PMuncertainty && PMreading.lastElement()<=PMthreshold+PMuncertainty){ activateVentilation=DecisionMaking(strategy, decisionMakingType, PMreading.lastElement(), PMuncertainty, PMthreshold);} \* if not in ambiguity zone verify if one or more pollutant levels are above safety thresholds and if it is the case activate ventilation. else if (COreading.lastElement()>COthreshold+COuncertainty || CO2reading.lastElement()>CO2threshold+CO2uncertainty ||VOCreading.lastElement()>VOCthreshold+VOCuncertainty || PMreading.lastElement()>PMthreshold+PMuncertainty){ activateVentilation=TRUE; } else{ \* calculate cumulative indices as in Equation (3) and IAQ index as in equation 5, if IAQ level is below comfortThreshold, calculate relative indices as in equation 6, referring to the last 15 min, and relative IAQ as in Equation (7). If relative IAQ is less than zero activate ventilation. alfaCOt = alfa0CO + alfa1CO*time+ alfa2CO*time^2; ICO = 100 − alfaCOt*log(cumulativeCO/cumulativeOptimalCO); alfaCO2t = alfa0CO2 + alfa1CO2*time + alfa2CO2*time^2; ICO2 = 100 − alfaCO2t*log(cumulativeCO2/cumulativeOptimalCO2); alfaVOCt = alfa0VOC + alfa1VOC*time + alfa2VOC*time^2; IVOC = 100-alfaVOCt*log(cumulativeVOC/cumulativeOptimalVOC); alfaPMt = alfa0PM + alfa1PM*time alfa2PM*time^2; IPM = 100-alfaPMt*log(cumulativePM/cumulativeOptimalPM); IAQ = min(ICO, ICO2, IVOC, IPM); if(IAQ < comfortThreshold){ ICOrelative = 100-alfaCOt*log(Integral(COreading, time, time-900)/Integral(outdoorCOreading, time, time-900)); ICO2relative = 100-alfaCO2t*log(Integral(CO2reading, time, time-900)/Integral(outdoorCO2reading, time, time-900)); IVOCrelative = 100-alfaVOCt*log(Integral(VOCreading, time, time-900)/Integral(outdoorVOCreading, time, time-900)); IPMrelative = 100-alfaPMt*log(Integral(PMreading, time, time-900)/Integral(outdoorPMreading, time, time-900)); relativeIAQ = min(ICOrelative, ICO2relative, IVOCrelative, IPMrelative); if(relativeIAQ < 0){ activateVentilation = TRUE; } } } if(activateVentilation==TRUE){ VentilationON(); wait(900s); VentilationOFF(); activateVentilation=FALSE; resetAllVariables(); } } } subroutine boolean DecisionMaking(strategy, decisionMakingType, measurementData, uncertainty, threshold){ \*chose between the two algorithms and call the specific subroutine if(decisionMakingType==“UtilityCostTest”){ **return** UtilityCostTest(strategy, measurementData, uncertainty, threshold);} else **return** FixedRisk(strategy, measurementData, uncertainty, threshold); }

**Listing A2.** Pseudo-code of Utility Cost Test algorithm
subroutine boolean UtilityCostTest (strategy, measurementData, uncertainty, threshold){ u[] = UtilitySetParameters(strategy); pdf[] = ProbabilityDensityFunction(measurementData, uncertainty) pA = Integral(pdf,threshold,-infty); p0 = (u[3] − u[2])/(u[0] − u[1] − u[2] + u[3]) if(pA > p0) **return** FALSE; else **return** TRUE; }

**Listing A3.** Pseudo-code of Fixed Risk algorithm
subroutine boolean FixedRisk (strategy, measurementData, uncertainty, threshold){ mra=FixedSetParameters(strategy); pdf[]=ProbabilityDensityFunction(measurementData, uncertainty) cumulativePdf[]= Integral(pdf); xmra=cumulativePdf.getIndexAtElement(mra); if(measurementData>xmra) **return** TRUE; else **return** FALSE; }
